# Criminalization of HIV non-disclosure: Narratives from young men living in Vancouver, Canada

**DOI:** 10.1371/journal.pone.0201110

**Published:** 2018-07-24

**Authors:** Rod Knight, Andrea Krüsi, Anna Carson, Danya Fast, Kate Shannon, Jean Shoveller

**Affiliations:** 1 British Columbia Centre on Substance Use, Vancouver, Canada; 2 Department of Medicine, University of British Columbia, Vancouver, Canada; 3 School of Population and Public Health, University of British Columbia, Vancouver, Canada; 4 Gender and Sexual Health Initiative, Vancouver, Canada; David Geffen School of Medicine at UCLA, UNITED STATES

## Abstract

**Background:**

Previous research has identified the impacts of legal frameworks that criminalize HIV non-disclosure among people living with HIV (e.g., elevated stigma and violence). However, far less is known about the perspectives or experiences of people–particularly, men–who are HIV-seronegative or who are unaware of their status. The objective of this paper is to describe the health and social risks that young men perceive to be associated with an HIV diagnosis in the context of Canada’s current legal framework pertaining to HIV non-disclosure.

**Methods:**

We analyzed data from 100 in-depth interviews (2013–2016) conducted with 85 young men ages 18–30 in Vancouver on the topic of the criminalization of HIV non-disclosure.

**Results:**

Our analysis revealed two dominant narratives in relation to HIV criminalization: (a) interrogation and (b) justification. An *interrogation* narrative problematized the moral permissibility of criminalizing HIV non-disclosure. In this narrative, Canada’s HIV non-disclosure legal framework was characterized as creating unjust barriers to HIV testing uptake, as well as impeding access to and reducing retention in care for those living with HIV. Conversely, a *justification* narrative featured a surprising number of references to HIV as a “death sentence”, despite effective treatments being universally available in Canada. However, most of those who presented the justification narrative asserted that the criminalization of HIV non-disclosure was morally justified in light of the perceived negative stigma-related impacts of HIV (e.g., discrimination; being ostracized from sex or romantic partners, friends, family). The *justification* narrative often reflected a belief that the legal framework provides both punishment and deterrence, which were perceived to supersede any barriers to care for both HIV-positive and -negative individuals.

**Conclusion:**

Public education regarding contemporary medical advances in HIV may help contest lay understandings of HIV as a “death sentence”, which is particularly relevant to destabilizing justification narratives. However, significant strengthening of HIV stigma-reduction efforts will be needed to move society away from narratives that attempt to justify Canada’s current HIV non-disclosure legal framework.

## Introduction

### Background

In Canada, the act of not disclosing one’s HIV seropositive status before engaging in sexual activities that pose a “*realistic possibility of HIV transmission*” can result in criminal charges, including aggravated sexual assault or attempted murder [1]. Few studies have examined how the criminalization of HIV non-disclosure influences public perceptions of HIV and HIV-related risks, particularly among those who are HIV-seronegative or who are unaware of their serostatus [2–4]. This knowledge gap is especially relevant for today’s generation of young men, who experience disproportionately high rates of HIV incidence (e.g., compared to previous generations of young men, older men). Today’s generation of young men also launch their sexual lives amidst a proliferation of novel advances in HIV testing, prevention and treatment interventions (e.g., Pre-Exposure Prophylaxis–PrEP; Treatment as Prevention–TasP). Some have argued that HIV criminalization, alongside these and other features of young men’s contemporary lives (e.g., social and sexual networking “apps”, evolving gender regimes), have changed the ‘rules’ of sexual engagement for young men, as well as their perceptions of HIV risk [5]. Yet, important questions remain about how the criminalization of HIV non-disclosure influences young men’s contemporary perceptions of and experiences with HIV risk.

### Canada’s HIV non-disclosure legal framework–an overview

Beginning in 1989 with the case *R v*. *Wentzell* [6], Canada began to see a small but growing number of cases in which an HIV-positive defendant was prosecuted for not disclosing their serostatus to a sexual partner. While the charges laid during this time varied (e.g., “administering a noxious thing”) [7], many of these early cases resulted in guilty pleas. However, by 1998, Canada’s first federal-level legal precedent involving the criminalization of HIV non-disclosure was established in the case of *R v*. *Cuerrier* in which the defendant–a man from the province of British Columbia (BC)–was convicted of failing to disclose his HIV-positive status to two women with whom he had had unprotected sex. Neither of the women were infected with HIV as a result of their sexual activities with Cuerrier. In this precedent-setting case, the Supreme Court of Canada ruled that people who are living with HIV are legally required to disclose their serostatus when there is a “significant risk of serious bodily harm”–regardless of whether or not HIV transmission occurs [8]. In the years following *Cuerrier*, various levels of the judiciary system interpreted the term “significant risk” differently. Rulings from many of the provincial courts, for example, regarded ‘failure’ to use condoms as a necessary element of “significant risk”, though this was inconsistently applied. For instance, in the case of *R v*. *Mekonnen* (2009), the Ontario Court of Justice convicted a man of two counts of aggravated sexual assault for failing to disclose his HIV status to female sex partners, despite having used condoms during vaginal intercourse.

In 2012, the meaning of “significant risk” was revisited by the Supreme Court during the appeal of two cases (*R v*. *Mabior* and *R v*. *DC*) in which it was ultimately ruled that people living with HIV must disclose their status before engaging in sexual activities that pose a “realistic possibility of HIV transmission” [9]. In part, this ruling was based on the premise that not disclosing one’s positive status in light of the “realistic possibility of HIV transmission” removes the opportunity for sexual partners to consent because they are unable to make a fully informed decision [10]. Similar to the “significant risk” ruling, the meaning of “realistic possibility” has been criticized for being overly vague and undefined, placing people living with HIV at risk for legal repercussions when engaging in sexual activities, including acts that are scientifically deemed to be of a low level of risk for HIV transmission (e.g., oral sex) [11].

Recent years have seen an increase in both the number of people charged for failing to disclose their status and the severity of the charges filed–a trend often referred to as “criminalization creep” [12]. For example, those who are accused of not disclosing their status can be charged with aggravated sexual assault or first-degree murder, both of which carry a maximum sentence of life imprisonment in Canada [13, 14]. To date in Canada, there have been an estimated 184 people who have faced prosecution of HIV non-disclosure in 200 cases since 1989 [15]. Many of these cases have received significant media attention, including several recent high-profile cases. In 2009, for example, in the case of *R v*. *Aziga*, the defendant was charged with 11 counts of aggravated sexual assault and two counts of first-degree murder after failing to disclose his HIV-positive status to 11 female sexual partners–seven of whom became HIV positive and two of whom died of AIDS-related complications. The case garnered significant media and public attention, as it was the first case in which an individual was charged with first-degree murder in connection with HIV non-disclosure in Canada. Both *Mabior* and *Aziga* were accused of employing “deceit and negligence” to spread the virus to unsuspecting sexual partners [9, 10].

While the Canadian HIV non-disclosure legal framework is ostensibly used to ‘govern’ the risk of HIV transmission, there is growing concern that the criminalization of HIV non-disclosure fails to prevent HIV transmission and instead increases overall harm [16]. For example, HIV non-disclosure legal frameworks have been implicated in increasing HIV-related discrimination [17], including the inequitable application of criminal charges among racialized individuals (e.g., Black and Indigenous people) [18, 19]. Recent research has also identified how HIV non-disclosure legal frameworks compromise patient-clinician relationships (e.g., trust, concerns about confidentiality) in clinical encounters [20]. Others have argued that the application of this framework undermines the conditions that are needed to promote the shared responsibility of preventing HIV acquisition or transmission (i.e., condom negotiation practices among sex partners) by placing the legal impetus on HIV-positive individuals, thereby contradicting decades of public health messaging that all individuals should take various actions to promote and protect sexual health [21].

There is also growing concern that the criminalization of HIV can have a set of unintended social consequences, including the exacerbation of HIV stigma. For example, previous research indicates that the criminalization of HIV non-disclosure is significantly associated with processes of stigmatization that serve to reduce access to regular testing [3, 11], in addition to linkage to treatment and long-term engagement with the HIV cascade of care. This has led some to suggest that the criminalization of HIV contributes towards a “syndemic of stigma” that both amplifies barriers to care while concurrently informing broader social attitudes and norms that legitimize and sustain the current legal framework in this area [22–24].

From a clinical perspective, others have argued that the current HIV non-disclosure legal framework has failed to fully consider that HIV is no longer a terminal condition in light of effective highly active antiretroviral treatment (HAART)–therapeutics that are universally available and in which the costs are covered by governmental programs within and across many global settings, including within the province of BC’s publically funded health care system. For example, the judicial application of the current ruling that a “realistic probability” of transmission does not seem to sufficiently or consistently account for the scientific consensus that “undetectable viral loads are untransmittable”–a consensus that is now firmly established, including through recent data published in 2016 from two large-scale clinical trials (PARTNERS and HPTN-052) [25–27] indicating that no HIV transmission occurred between serodiscordant sexual partners when the person living with HIV was on treatment and had an undetectable viral load [27].

Previous research has identified the impacts of legal frameworks that criminalize non-disclosure on people living with HIV (e.g., elevated stigma and violence) [28]. But, far less is known about the perspectives or experiences of people, who are HIV-seronegative or who are unaware of their status. Moreover, little is known about the perspectives of men, particularly young men, including within Canada’s current HIV non-disclosure landscape. The ways in which they perceive Canada’s HIV non-disclosure legal framework is important for several reasons, including how it reveals the current generation’s understandings about HIV risk, their perceptions about the social and health impacts (intended and unintended) of the framework, as well as the enduring power of HIV stigma.

### Young men, an evolving HIV intervention ‘landscape’ and the criminalization of HIV non-disclosure in Canada

The concurrent proliferation of novel advances in HIV testing and effective antiretrovirals alongside the high and rising prosecutorial rates for HIV non-disclosure [19, 29] within Canada contributes to a vastly different contextual ‘backdrop’ for today’s young men than for previous generations. Some have argued that these and other features of young men’s sexual lives (e.g., ubiquity of social and sexual networking apps, evolving gender regimes) have changed the rules of sexual engagement for young men, including contemporary understandings of HIV-related risks [5, 30]. For example, theoretical and empirical work in this area reveals that young men’s understandings of the social and health outcomes of an HIV diagnosis are deeply connected to features of social context, including the socio-legal implications of the HIV “criminalization creep” [30–33]. Yet, few studies have examined how the criminalization of HIV non-disclosure, alongside evolving intervention ‘landscapes’, influence young men’s perceptions of and experiences with HIV-related risk–particularly among those who are HIV-seronegative or who are unaware of their serostatus. At this juncture, we suggest it is important to identify how today’s young men situate their understandings of HIV within Canada’s highly criminalized context regarding the non-disclosure of HIV. The objective of the current analysis is therefore to identify the narratives used by young men as they rationalize or challenge the criminalization of HIV non-disclosure.

## Methods

For the reader, it is helpful to consider our motivations for conducting an analysis on young men’s perspectives of HIV non-disclosure legal frameworks. The current analysis was conducted within a larger program of research identifying the social and structural determinants of young men’s sexual health, with particular attention on how Vancouver’s evolving HIV intervention ‘landscape’ influences young men’s sexual health experiences. Within this program of research, a key aim is to identify how various features of contemporary socio-cultural contexts (e.g., norms about young people’s sexual health practices) may contribute to barriers to HIV care, including, for example, how legal frameworks regarding HIV non-disclosure influence young men’s understandings about HIV risks. We employ narrative inquiry [34, 35] to analyze 100 interviews with young men (ages 15–30) which elicited their perception of the criminalization of HIV non-disclosure. Narrative inquiry focuses on the stories told (e.g., challenges, obstacles and resolution) by participants, while concurrently positioning stories as possessing a central role in identity formation (e.g., of self and others) [36–38]. This approach to narrative inquiry is founded on the premise that people tell stories as a way to work through and acknowledge the meaning of events and also provides a means to reflect upon how an individual incorporates their social and normative understandings into their lived experiences, including how they justify or contest various norms, events and outcomes [34]. In our analysis, we pay particular attention to how young men’s stories are told in order to identify the social and normative meanings that underpin their various perspectives regarding Canada’s HIV non-disclosure legal framework [34, 39].

### Study setting

In Canada, HIV prevalence is 212 per 100,000 population, with an estimated 65,040 Canadians living with HIV [40, 41]. Data were collected in Metro Vancouver in the province of BC, Canada, between 2013–2016. The region’s total population in 2014 was 2,474,123, including 131,418 young men between the ages 18 to 30 [42]. In line with the historical trends in BC, men continue to have higher rates of HIV acquisition than females [43]. In 2014, the HIV incidence rate was 14.6 cases per 100,000 among young men ages 20 to 24; and 19.7 cases per 100,000 among young men ages 25–29 (compared to the provincial average of 9.4 cases per 100,000) [43]. The BC Center for Disease Control (BCCDC) reports that 57.5% of all new HIV diagnoses in BC occurred among gay, bisexual and other men who have sex with men (MSM), 1.3% of whom concurrently reported injection drug use [43]. In BC, Indigenous people are disproportionately affected by HIV [43]. In 2014, Aboriginal peoples comprised 11–17% of new HIV diagnoses in the province, while representing only 5% of the total population [43]. The HIV incidence rate in 2014 was 27.3 cases per 100,000 among Aboriginal men (and 19.6 cases per 100,000 among Aboriginal women), exceeding the provincial rate of 9.4 per 100,000 (and 1.9 cases per 100,000 among Aboriginal women) [43].

### Interviews

This study draws on 100 in-depth, semi-structured interviews with 85 young men ages 15–30. We used a stratified purposive sampling strategy to deliberately recruit young men from a variety of sociocultural backgrounds and lived experiences [44], including young men who may experience vulnerabilities regarding the health and social impacts of STI/HIV. Participants were recruited to participate in the study through online advertisements (e.g., Facebook; Craigslist), and advertisements located at clinical (e.g., posters at youth sexual health clinics) and non-clinical settings (e.g., youth centers; bus stops). Participants also were recruited from the At-Risk Youth Study (ARYS), a prospective cohort of youth living in Vancouver who are or have previously been street-involved and used illicit drugs (other than marijuana) (see Wood et al. [45] for more details regarding the ARYS cohort). Eligibility criteria included: being between the ages of 15 to 30; ability to speak and understand English; identifying as a man (including cisgender or transgender men); and currently or previously having been sexually active. We did not ask participants to disclose their HIV serostatus, a decision we arrived at after considerable discussion and debate within our team.

Participants received a CDN$30 honorarium to compensate them for their participation. Ethics approval for the study protocol was obtained from the University of British Columbia’s Behavioural Research Ethics Board (#H12-01936). Further details regarding our recruitment and interviewing procedures and protocols have been published elsewhere [46]. This approval allowed participants ages 15–18 to participate as emancipated minors, meaning they did not require parental or guardian consent to participate in our study.

Participants completed an informed consent form and a 9-item socio-demographic questionnaire prior to participating in an in-depth, semi-structured individual interview. All interviews were conducted within our team research offices at UBC and downtown Vancouver by co-authors AC and RK. The interviews lasted 1 to 1.5 hours in duration and were audio-recorded and transcribed. Participants were asked to share what they know about the legal framework surrounding HIV non-disclosure in Canada. As our interviews progressed, we provided additional background information about the legal framework, including a brief description of the legal status of HIV non-disclosure in Canada (see Table 1 for a short script the interviewers provided to participants). We also offered participants the opportunity to ask questions of clarification regarding Canada’s HIV non-disclosure legal framework. We then invited participants to discuss how their understandings of HIV and HIV risk might relate to the broader context of Canada’s current legal framework pertaining to HIV non-disclosure. The bulk of the interview focused on questions that elicited participants’ perspectives on whether they feel Canada’s HIV non-disclosure legal framework is justifiable or not.

**Table 1:** Interview script regarding information about Canada’s HIV non-disclosure legal framework.

In Canada, we have a legislation that criminalizes HIV+ individuals who do not disclose their HIV + status to sexual partners unless they use a condom AND have a ‘low’ viral load. **During this section, I am not asking about what you have done in the past, but am interested in understanding your opinions on this issue.**
a. Have you come across any information related to this legislation? Tell me about that.
b. In Canada HIV non-disclosure has most often been prosecuted as aggravated sexual assault. Aggravated sexual assault carries a sentence of jail time up to a maximum of life imprisonment and registration on the Sexual Offender Registry. This is one of the most serious crimes in the Canadian Criminal Code. In Canada, over 150 people have been prosecuted for HIV non-disclosure to date, even where no transmission has taken place and where in many cases the risk of transmission was considered to be very small. What are your initial thoughts on how HIV is treated in Canada in these sorts of circumstances?

### Analysis

In order to identify the narratives that young men used to describe their understandings of HIV and Canada’s HIV non-disclosure legal framework, we began by coding all interview data thematically using NVivo 10, at which point we read and reread the young men’s accounts of the criminalization of non-disclosure legal framework. Discrepancies between codes, coders, emergent themes and co-author interpretations were resolved through discussion and re-visiting the raw data during team meetings. Memos were kept by coders to document the analysis process as it developed, and these were discussed with the co-authors throughout the analysis of the data and writing of this article. We also used verification strategies to advance a consensus-driven representation of our findings, including providing opportunities for all authors to adjust or revise and/or respond to the presentation of each thematic as presented in the manuscript. Thus, the central narratives that emerged through our second set of readings were brought to team meetings and discussed in an iterative fashion, during which we reflected on how specific narratives associated with the criminalization of HIV non-disclosure fit within young men’s broader interviews and the broader set of interviews (a holistic-content analytic approach to narrative inquiry [47]).

After the central narratives were identified, we reread the young men’s stories with particular attention paid to *how* the stories were told, and how their stories reflected and incorporated various features of social context (e.g., socio-cultural norms regarding young men’s sexuality) in which these stories unfolded [48]. For example, the voice in which young men spoke (e.g., their own, that of others) and the specific aspects of the legal framework that were (de)emphasized were examined. We paid particular attention to the details that were expressed in a more emotionally responsive manner (e.g., with anger, with fear for self or of others) by returning, when necessary, to our audio recordings and interview notes. In this way, we gained an enhanced understanding of the resources (e.g., social/interpersonal, educational, other forms of knowledge) young men drew upon in the construction of their own personal accounting of Canada’s legal framework surrounding HIV non-disclosure, as well as the ‘sense-making’ processes (i.e., rationalizing or challenging) young men engaged with when expressing their perspectives regarding the criminalization of HIV non-disclosure during interviews. As such, our analysis includes both a consideration of the structure (i.e., how participants told stories) and content (i.e., the information presented within stories) of the narratives expressed by the study participants.

## Results

### Study participants

A total of 100 interviews were conducted with 85 participants, of which 28% (*n* = 24) identified as gay, 9% as bisexual (*n* = 8), 55% as straight (*n* = 47) and the remaining 5% as pansexual (*n* = 2) or other (*n* = 4). Forty-eight percent (*n* = 24) were recruited from the ARYS cohort, with the remaining 52% (n = 26) recruited either through online advertising, posters or word of mouth. A total of 5 (6%) participants had not been previously tested for HIV. Table 2 provides additional socio-demographic information.

**Table 2:** Socio-demographic characteristics of study participants.

**Ethnicity**^**a**^	(n)	(%)
Indigenous	17	20
Black	5	6
Latin	6	7
Middle Eastern	3	4
South East Asian	16	19
White	51	60
Other	2	2
**Living Arrangement**		
Alone	13	15
Friends/roommates	27	32
Parents/family	15	18
Partner/spouse	9	11
Shelter/on street	17	20
University residences	3	4
Other	1	1
**Age**^**b**^		
15–19	7	8
20–24	61	72
25–30	17	20
**Sexual Orientation**	(n)	(%)
Bisexual	8	9
Gay	24	28
Heterosexual/straight	47	55
Pansexual	2	2
Other	4	5
**Gender Identity**		
Transgender man	1	1
Cisgender man	84	99
**Recruitment medium**		
ARYS	41	48
Craigslist	3	3
Facebook	31	37
Poster	5	6
Snowball (i.e., word of mouth)	1	1
Community-based ASO	4	5

^a^15 participants indicated 2 ethnic identities.

^b^Mean age = 23

### Overview of findings

During the interviews, we solicited participants’ perspectives on the legal framework that criminalizes HIV non-disclosure in Canada. Approximately half of all participants either did not have previous knowledge about the criminalization of HIV non-disclosure in Canada or knew very little about the specifics of the legal framework; all participants were provided with the description of the law detailed in Table 1. Below, we present our findings within two sections pertaining to the two dominant narratives in relation to participants’ perspectives on HIV criminalization that arose within our interviews: (1) *interrogation* and (2) *justification*. Quotations from participants’ transcripts are presented to illustrate the various themes that arose within each narrative. Each quotation is preceded by a short description of each participant’s socio-demographic profile and followed by a researcher-assigned numeric code.

### Justification narrative

Both participants who were and were not previously aware of the legal framework regarding HIV non-disclosure prior to our interview *justified* the framework. Some participants did so on the basis that they equated an HIV diagnosis with a “death sentence”. Several participants told us that an HIV diagnosis would result in the rapid decline of health (e.g., progression to full-blown AIDS). Within this narrative, the physical act of sexually transmitting HIV was described as being analogous to other forms of physical assault (e.g., “stabbing”) and even murder, and participants frequently drew on cases that they had heard about from within various media sources. These narratives employed a lexicon that portrayed HIV non-disclosure as inherently aggressive, violent and intentional. For example, one 22-year-old and one 19-year-old straight man each explained:

I have to agree with [the criminalization of HIV non-disclosure]. Like, fuck, absolutely. I mean that’s worse than stabbing someone. At least that person is just gone at that moment if they’re stabbed. (041b)

Well, if you didn’t disclose that to someone then you’re pretty much putting a death sentence on them. I mean, they might have a while to live but you’re infecting them with a deadly disease. Legal actions should be taken towards that. (005)

A more dominant justification narrative rationalized the criminalization of HIV non-disclosure by pointing to the perceived *social impacts* of unknowingly acquiring HIV from a partner. These participants described a variety of devastating personal and social consequences that they felt they themselves would experience if faced with an HIV diagnosis, including suicidal ideation, the loss of current and future sexual and romantic partners and experiences, extreme isolation (e.g., from family and friends), and systemic discrimination from society broadly. For example, one 23-year-old and one 22-year-old straight man each reflected on how they viewed the legal framework as being justifiable because they felt their lives would change for the worse if they were to become HIV positive in the event that a partner did not disclose their HIV-positive status:

I mean, if I had HIV I would definitely… Wow. That’d be difficult, you know? I would probably like, like I said, I would probably want to kill myself. I’d probably start using drugs to numb the pain. (046)

I would be infinitely more cautious and perhaps even abstinent to some extent. Cause I don’t think I could deal with the guilt of potentially passing it on to someone. So that would really, I mean, affect that aspect of my life. It’d maybe, to some degree, eliminate that aspect of my life. (016)

These participants tended to justify HIV non-disclosure legal frameworks by focusing on the perceived health or social consequences that they feared they would experience if they were to become HIV positive as a result of HIV non-disclosure. These participants argued that having sex with someone who did not disclose their HIV-positive status would *de facto* result in the transmission of HIV, thereby presupposing a ‘uniformity of risk’ with regard to the sexual transmission of HIV.

Another sub-set of participants focused less on the consequences of acquiring HIV and more on the moral dimensions that they associated with the *act* of an HIV-positive person not disclosing their status to sex partners. For these participants, many understood that the risk of transmitting HIV could be extremely low, including, for example, in cases where an individual has low viral loads or undetectable levels. Nevertheless, these participants felt that the legal framework was justified regardless of the level of risk involved. For example, two 23-year-old straight men described:

It doesn’t matter if transmission took place or not, or what that person’s viral load was. The point is that person has a very serious life-threatening disease, and that information was not shared to a person who could’ve come in direct contact through the best known transmission method of that disease and, you know, like- if it was by chance that that disease wasn’t transmitted–if it had been, that person would’ve been in trouble. When it comes down to it, that information needs to be shared first and foremost. (036b)

If you set the precedent of, “Well, you know, they didn’t get AIDS…” then … how did you know that they wouldn’t? I mean, the “Well-it-didn’t-happen-so” approach isn’t the way to approach something like AIDS. (012b)

As participants reflected on the act of not disclosing an HIV positive status to sex partners, a series of highly sensationalized accounts emerged in which participants began to construct extremely ‘villainous’ characters with nefarious qualities and whose actions and motives would be inherently malicious and secretive, and several referred explicitly to details about cases they had read about previously. As they did so, participants tended to position HIV-positive individuals as being the antagonist in their narratives–someone who premeditates the act of not disclosing their HIV-positive status in order to cause harm to innocent and unsuspecting partners. As such, these narratives tended to present scenarios that ‘required’ the legal framework to respond accordingly. For example, upon hearing about the legal framework for the first time, one 22-year-old Two-Spirit participant suggested the legal framework was helpful in preventing others from purposefully connecting their “enemies” with HIV-positive sex partners so they could become infected:

Well, I don’t know much about it [HIV non-disclosure legal framework], but I think it’s really good cause I know that there’s some really messed up people out there and there’s like websites where you can hook an enemy up with someone so that they get infected with AIDS. So it’s like, a easy way to shorten someone’s life, by hooking them up with someone who’s a positive and has sex with them or seduces them or whatever. (047)

All of the participants who justified the legal framework emphasized that they felt there was value in having an HIV non-disclosure legal framework in place because it would: (i) *deter* HIV transmission from occurring by discouraging those living with HIV to have sex without disclosing via the threat of a severe punishment and retribution; or (ii) *punish* those who did not disclose their seropositive status to their partner(s). For example, one 23-year-old straight participant described:

I think this is a way that might make this person think twice before having intercourse with his partner or her partner and not let them know that they have HIV. […] I think it’s good because it kind of teaches a lesson, but it also kind of scares the other people to not do this. Because of the legislation. (008)

There were two other kinds of arguments that arose within the justification narrative that we identified in our analysis. First, a justification narrative emerged in which participants suggested that the law–in its current form–did not “go far enough”. A few participants offered additional strategies that they felt would help prevent HIV transmission from occurring. For example, a 21-year-old straight man described:

I was also thinking about maybe, like have a sign on these people. Like make them wear something to let the public know when they interact with these people. I mean, I really want to stop it [HIV] as much as possible. Trying to keep it to themselves is not going to resolve someone’s life. I really don’t believe that. I think the risks are going to be taken and they–if I was in the authority, I design a sign and have them put it on their body. More than just that, something that’s not removable. And when these people interact with these people, then they can recognize the sign and then stop them from what they’re doing. (001)

Conversely, among a sub-set of participants, a dissonance began to arise in which they started questioning how the various pros and cons that they associated with the legal framework should be balanced in order to produce more socially just outcomes. This justification narrative tended to be underpinned by a recognition that there can be highly nuanced, personal and other contextually contingent issues with regards to various cases of non-disclosure that would be important to take into account when making case decisions. For example, one 22-year-old straight man began interrogating the various elements of the legal framework–though he ultimately felt the legal framework was necessary to protect people against those individuals who were purposefully transmitting HIV to others:

But a lot of them are like, decent people, right? They wouldn’t want to infect somebody with HIV. It’s not really something you wanna do. But some people might feel like “Oh, I have HIV so now I wanna spread it because I feel so shitty about having it and I wanna hurt other people.” Some people do think that way. But not everybody, right? (027)

### Interrogation narrative

As in the justification narrative, both those who were and were not previously aware of the legal framework prior to our interview *interrogated* the framework, and the interrogation narrative were generally founded on the premise that the application of the legal framework does not result in justice. Some participants perceived that the framework does not sufficiently reflect today’s context of universal treatment and effective HIV prevention strategies (e.g., HAART; PrEP). For these participants, a *biomedicalized* view of HIV that focused on the ‘technical’ features of HIV transmission (e.g., “realistic probability of transmission”) was prioritized and juxtaposed to HIV stigma. For example, one 23-year-old straight man interrogated how the risk of HIV transmission is not uniform:

It requires a bodily fluid transfer, and the transfer actually has to be pretty substantial to catch it. Like, if I accidentally got the saliva of somebody with HIV in my mouth, I wouldn’t necessarily contract HIV. So, the entire stigma around it is just ignorance on the part of people who have the stigma. It’s like, it’s a disease. A virus. A disease. It’s just like cancer is a disease. People have it. It doesn’t mean you’re going to get it. Doesn’t change who the person is. It’s just something that they’re going to have to deal with. They’re going to take their medication, and hopefully they’re going to level out. (012)

Participants challenged the moral justifiability of criminalization of HIV non-disclosure because they felt the current HIV prevention ‘landscape’, including universal access to HIV prevention, treatment and care has brought HIV into the realm of a chronic condition that no longer infers a “death sentence”. These participants expressed frustration that HIV is treated ‘differently’ from other infectious diseases. For example, one 23-year-old gay man described how, in the context of effective treatment opportunities, he considers going for HIV testing as more of a regular health “check-up”:

I moved to the thought that, you know, HIV and AIDS is just like the common cold and they’re just something else you can have. Whereas, I used to think it was like ‘This is the end of the world’ versus, you know, ‘It’s just out there with anything else you can even have.’ […] I think it’s just like the common cold! […] HIV does not equal AIDS. (026)

Another sub-set of participants interrogated the legitimacy of the legal framework as they felt that, in practice, the framework would be difficult to apply in ways that could sufficiently take all of the evidence into account, particularly with regards to potentially conflicting reports from each party involved. Several participants recognized the power dynamics involved in the negotiation of sex, and these participants tended to underscore the complex relational dimensions of this process and how it could be difficult to translate how this occurs within a court room. For example, one 23-year-old gay man described:

Unless you video record, it’s a “he said, she said” at the end of the day. But, so again, that’s not the fact that somebody gives [HIV to] you, you have bigger concerns than that. You know, it’s a “he said she said”. If they said “Oh, I told you”, you said “I didn’t …” who are you gonna believe? There’s no–it’s not–it’s not that easy, especially in that situation of–there’s probably a relationship involved there. Even if it’s just a one-night stand, there’s a–there’s a human relationship there. The personal and intimate relationship, so it’s not as easy as “I robbed a guy” or “I hit your car”, let’s say. Evidently, there were witnesses and cameras. That’s–that’s an easy one. If it’s behind closed doors and it’s the whole thing where the government has nothing to do in your bedroom and–I mean how–I don’t know how people could prove that. (026)

Another sub-set of participants suggested that a critical component of the legal framework would be the need for those living with HIV to be fully informed about the legal framework in order for it to be just or effective. Some of these participants began to suggest there could be reasonable scenarios in which somebody would not be able to fully appreciate the importance of disclosing their HIV-positive status (e.g., due to a limited cognitive capacity). Others suggested that there could be scenarios in which an HIV-positive individual does not know enough about the legal framework, and that this would delegitimize the overall reasons why the framework is being implemented (e.g., to deter or punish the act of non-disclosure). For example, one 21-year-old straight man described:

There’s wiggle room for like things to get lost in translation type of thing, so in that case where someone has sex with someone else and they’re positive, and the person that they had sex with is negative and then they didn’t tell them…then they’re charged with it and they say, “But I didn’t know, like how was I supposed to know ‘cause I’ve never like been exposed to the legislation and I thought somehow…” Let’s say they’re from like the northern territories like in the Yukon or something where there isn’t like ready–where there isn’t like Internet access or anything like that… they have no access to the actual constitution where the legislation is written, then it becomes tricky because it’s like, they didn’t know about it. Are we still gonna charge them like with like the full punishment even though like they’ve proven that they actually had no way of knowing about the legislation?” (009)

As participants reflected on the legal framework regarding HIV non-disclosure further, a narrative also emerged in which the framework was positioned as being more of a socially constructed phenomenon, rather than one rationally informed by careful reasoning and the current realities of HIV risk and prognosis. These narratives frequently situated the legal framework as being structurally embedded within the socio-historical context of HIV more broadly. For example, one 20-year-old gay man argued:

I don’t think I know of any diseases that have like criminal liability associated with them. Like, what’s so special about this disease? Is it because it’s drug users? Is it because it’s sex? It’s this idea that there’s something different about this virus. It’s completely socially constructed. (014)

Despite recognizing that the health effects of an HIV diagnosis were not as severe as they had once believed it to be, all of the men in our study who interrogated and questioned the legitimacy and justification surrounding the HIV non-disclosure legal framework said that testing HIV positive would significantly affect them socially. For example, despite previously describing that the health-related effects of HIV would be manageable, one 21-year-old gay participant began telling a story about how his friends and family might treat him differently if he were HIV positive:

I think everyone around you would treat you differently. Naturally, some positive, some negative, in the sense that, I guess, I think there would be a lot of pity. For the majority of the other people who understand like, it’s not the end of the world, but the general public who doesn’t get it. […] It’s like you’re wearing that like, hood of shame. But like, there’s, you know, less reason for it today because of medicine, so, like again, I think it’s like that interplay between how you personally deal with it and then how society treats you. […] So … I don’t think the law takes that into account the very personal, secular nature of it. (023)

Despite recognizing that there could be profound social impacts on their lives if they were to unknowingly acquire HIV, those who articulated an interrogation narrative continued to question the legitimacy of the framework. For example, many of these participants suggested that a legal framework that seeks to punish HIV-positive individuals would invariably lead to the further exacerbation of HIV stigma. These participants frequently described how they felt the framework was not capable of producing justice and was also counterproductive from a public health perspective. For example, these participants problematized the criminalization of HIV non-disclosure as creating barriers to effective HIV prevention and care strategies. These concerns were particularly salient among participants who viewed themselves as being at ‘elevated’ risks for HIV, including many of the gay and bisexual men and those with histories of injection drug use. Their narrative underscored how the criminalization of HIV non-disclosure presents barriers to effective testing treatment and linkage to care strategies by: (i) waylaying opportunities to advance the public’s understandings about HIV and HIV risk; and (ii) further perpetuating HIV stigma. For example, one 22-year-old bisexual participant described:

Just having a law like that with something like HIV is just, playing into a problem that would just make people feel less likely to volunteer to go get tested or screened. It feels like it is just playing into the stigma of the illness rather than trying to actually help victims of people who have taken advantage of them by not telling. This does not create a dialogue. (018)

Several participants concluded their interviews with us by describing how they were unsurprised that an HIV non-disclosure legal framework existed in Canada. For these participants, they viewed the framework as a reflection of an unjust society, rather than as a mechanism to produce fair outcomes in the criminal justice system. For example, one 24-year-old gay man described:

Somehow I cannot really blame the law because the law is more of a reflection of the society. If society stops progressing then there is kind of no point in changing the law. And if society is progressing the law would change to adapt to the society. I don’t think the law is the main problem. It’s the public education, the public perception on HIV positive people about sex and education–that it’s about communication and human interaction. I don’t think it’s the mistake of the law. No. The law will change itself when people ask for it. And when people understand that everything’s going to be managed. (069)

## Discussion

Our findings revealed two narratives in relation to HIV criminalization: *justification* and *interrogation*. Within the *justification* narrative, participants asserted that criminalization approaches could be justified because they reflect the gravity of the perceived health or social consequences of unknowingly acquiring HIV from an HIV-positive partner via processes associated with HIV stigmatization (e.g., status loss, discrimination, isolation). Of note, all participants who articulated a justification narrative focused on how the legal framework could protect themselves (e.g., as HIV-seronegative men), rather than how the legal framework could impact those who are HIV-seropositive. Here, the justification narrative tended to be deployed ‘out of context’ (e.g., without taking into account the current HIV intervention ‘landscape’); for example, participants tended to assume that having sex with someone who did not disclose their HIV-positive status would inevitably result in the transmission of HIV. These arguments also tended to be founded on a fear that HIV-positive individuals would purposefully transmit the virus. As such, these participants felt the legal framework was justifiable as a *deterrent*.

Conversely, the *interrogation* narrative tended to problematize the idea of criminalizing HIV, and this narrative was embedded within far clearer understandings about today’s HIV intervention ‘landscape’. First, this narrative emphasized how universal access to HIV prevention, treatment and care has brought HIV into the realm of being a chronic condition, rather than a “death sentence”. Secondly, the application of a legal framework that criminalizes people living with HIV was characterized as creating barriers to HIV testing uptake, as well as impeding access and reducing retention to care for those living with HIV. Finally, the justification narrative situated the legal framework as being complicit in–and even a structural driver of–HIV stigmatization, and thereby counterproductive to justice and to improving population health.

Despite the universal availability of HAART in the Vancouver setting and decades of public health education surrounding HIV, the persistent discourse about HIV “death sentences” reveals the extent to which today’s generation of young men may not embody the treatment optimism so often ascribed to them [49–51]. The justification narrative also drew strongly on HIV stigmatization. At this juncture, we suggest that future research regarding risk-mitigation and behavioural disinhibition ought to focus more on the *social* impacts of HIV and HIV risk. Understanding how HIV stigma is embodied in the behaviour of today’s generations of young men may provide insights into improved public health messaging HIV and HIV risk (e.g., current treatment realities; PrEP) [52].

We were struck by the extent to which the justification narrative tended to be offered by respondents with relatively ‘low’ HIV risk profiles and/or who identified as straight. For example, within our sample, very few of the gay or bisexual participants advanced a justification narrative and these tended instead to be put forward by straight participants. In part, this could be related to HIV as continually being represented as largely an issue for gay and bisexual men, while not being as important for other men–divisions or siloes along so-called “sexual cultures”. We also noted that the justification narrative drew on many of the discourses that are used to justify the legal framework as an effective means to “govern” HIV risk–discourses that are frequently advanced by media depictions and by legislators in favour of this approach [53]. First, a set of consequentialist arguments tended to assume that the overall effect of the legal framework would be protective from a public health perspective–a claim that runs counter to the growing body of evidence in this area that indicates overall harm is enhanced through the criminalization of HIV non-disclosure [19, 29]. In so doing, the justification narrative tended to also disregard how various features of the legal framework may serve to disproportionately exacerbate health inequity, particularly among already marginalized populations. As a result, the justification narrative tended to over-emphasize individual-level lifestyle factors (e.g., agentic capacity to negotiate safe sex) as being the primary driver of HIV risk–another claim that also runs counter to the decades of social science, epidemiological and behavioural science that identifies structural drivers of HIV (e.g., laws, legislation, socio-cultural norms) as constituting the most influential determinant of HIV risk [23, 54].

In Canada, the current federal government has expressed interest in advancing more socially just responses to the criminalization of HIV non-disclosure [55]. We concur there is an urgent need for legal reform and that appropriate reforms may be especially important for how young men embody understandings about HIV in their HIV-related behaviour (e.g., negotiation of safe sex; prejudicial understandings of people living with HIV). Based on the results of our study, we argue that changes in the HIV non-disclosure legal framework need to take into account how ‘punish-’ and ‘deter-based’ approaches to “governing” HIV risk feed into (and are reflected by) unsympathetic moral opinions and prejudicial understandings of people living with HIV that, ultimately, exacerbate HIV stigma.

Our findings also underscore the extent to which Canada is currently presented with the opportunity to implement a rare *structural-level* intervention that can have real and long-lasting effects on the underlying cultural and social conditions that cultivate HIV-related attitudes and behaviour among key populations, including young men. As an example, the Canadian Department of Justice has released a report acknowledging that HIV is primarily a public health issue [1]. Nevertheless, we recognize the complexity of advancing new legislation in this area and/or the advancement of prosecutorial guidelines (see the Canadian HIV/AIDS Legal Network’s 2017 report for a discussion on the complexities involved in this process) [56]. It is our hope that future changes to the HIV non-disclosure legal framework (e.g., provincial and federal prosecutorial guidelines; legislative reform) will be advanced in ways that more fulsomely reflect the contemporary realities of HIV treatment and prevention while concurrently intervening on narratives that are insensitive to community norms (e.g., the negotiation of safer sex, gender relations) and fail to advance justice. Indeed, involving community perspective in efforts to reform the legal framework will be integral to success.

This study has strengths and limitations. First, we acknowledge that narratives are highly context-dependent (e.g., stories told to an interviewer are influenced in terms of both form and content and may differ from the stories that are told to young men’s peers) [57]. Young men’s narratives are likely far more fluid and dynamic than our current analytic approach has allowed us to capture. Second, while the composition of our sample was intended to reflect a diversity of young men, it is not intended to be ‘representative’ of all young men living in Vancouver. Third, our findings provide insights from a diverse set of young men, including those who voluntarily revealed that they are HIV-seronegative or those who revealed that they do not know their HIV serostatus; no participant revealed that they were living with HIV. For ethical and legal reasons, we did not ask participants to disclose their HIV-serostatus. (As an aside, during the review process, we were reminded of the irony that the very issue we have sought to study–HIV non-disclosure legal frameworks–resulted in a key limitation of our study design, i.e., our inability to discuss serostatus in the context of HIV risk behaviour.) Therefore, we do not know the serostatus of all participants. It is possible that a sample comprised solely of HIV-seropositive men, for example, may have resulted in a different set of narratives regarding the criminalization of HIV non-disclosure. Fourth, future research in this area should identify how HIV-related behaviour (e.g., HIV testing, condom or PrEP use) is influenced by the HIV non-disclosure legal framework. Finally, while, towards the end of our data collection activities new insights were no longer generated regarding HIV non-disclosure (thereby indicating theoretical saturation was attained), the findings are not claimed as ‘representative’ or generalizable to all young men. Nevertheless, including a diverse set of young men (e.g., men of various sexual identities) in our analysis helped us to uncover the influence of broad social norms on young men’s understandings about HIV and HIV risk as they rationalized or challenged the criminalization of HIV non-disclosure.

### Conclusion

Our findings reveal how community-situated narratives that justify the criminalization of HIV non-disclosure do so in ways that both draw on and reinforce HIV stigma, rather than in ways that emphasize justice or the realities of contemporary HIV intervention ‘landscapes’. Public education regarding contemporary medical advances in HIV may help contest lay understandings of HIV as a “death sentence”. However, significant strengthening of HIV stigma-reduction efforts will be central to the further deconstruction of narratives that disregard justice in favour of the punishment and deterrence of a historically marginalized population. Critically, reforms to the current legal framework will be among the most effective strategies to have a large reach and long-lasting effect on the underlying cultural and social conditions that cultivate insensitive moral opinions and prejudicial understandings of HIV.
